# Using Social Media to Communicate Child Health Information to Low-Income Parents

**Published:** 2011-10-15

**Authors:** Stephanie J. Stroever, Michael S. Mackert, Alfred L. McAlister, Deanna M. Hoelscher

**Affiliations:** Michael & Susan Dell Center for Healthy Living, The University of Texas School of Public Health, Austin Regional Campus; The University of Texas at Austin, Austin, Texas; The University of Texas School of Public Health, Austin Regional Campus, Austin, Texas; Michael & Susan Dell Center for Healthy Living and The University of Texas School of Public Health, Austin Regional Campus, Austin, Texas

## Abstract

The objective of this study was to determine the value of using social media to communicate child health information to low-income parents. We evaluated qualitative data obtained through focus groups with low-income, predominantly Hispanic parents. Results were mixed; lack of time and credibility were the primary objections parents cited in using social media to obtain information about their children's health. Social media has value as part of an overall communication strategy, but more work is needed to determine the most effective way to use this channel in low-income populations.

## Objective

Academic researchers who study child and adolescent health have a responsibility to disseminate their research to parents in ways that can facilitate behavior change. Research on the most effective way to communicate health information to parents, especially among low-income parents, is limited (1-4). The Internet and its related information-sharing applications, specifically Web 2.0 social media, are innovative methods for communicating child health information to parents. Our primary objective was to collect and analyze qualitative data to determine whether social media is a valuable (ie, worthy investment of health promotion resources) and effective way to communicate health information to low-income parents in an effort to promote children's health.

## Methods

We selected participants through homogenous, purposeful sampling by using standard recruitment guidelines (5). Inclusion criteria were 1) being a parent of a child who attends a school in a designated central Texas school district and 2) being English-speaking. Students who attended these schools were predominantly Hispanic (79%) and economically disadvantaged (81%), as determined by eligibility for public assistance (6-7). We distributed flyers with information about focus groups at a school-based children’s health clinic, which is the only source of pediatric care in the area and which serves primarily low-income, Hispanic families (8).

**Table:** Box. Questions Used to Prompt Discussion During Focus Group Study, Texas, October 2010

Are you familiar with social media? Do you use social media websites? What do you like to do on them?
Would you consider using social media applications, such as a Facebook fan page or blog as a way to get information about your child's health? Why or why not?
What if these social media sites were from a university, government agency, or nonprofit organization? Would you use them to get information about your child's health?

Study approval was obtained from the University of Texas Health Science Center at Houston institutional review board, and voluntary informed consent was obtained. We conducted 4 focus groups (N = 19; mean no. of participants, 4.75; range, 2-6 participants) at public libraries in October 2010. The groups lasted 45 to 60 minutes and included a discussion, prompted by questions (Box) on the use of social media and willingness to use social media as a health information resource. Participants each attended only 1 group and were provided an incentive for their time.

We collected demographic information and administered the English version of Newest Vital Sign, which determines the likelihood of low health literacy. The test is reliable and correlates with the Test of Functional Health Literacy in Adults (TOFHLA) (9). We recorded and transcribed each group's comments for analysis with the classic analysis strategy described by Krueger and Casey (5). Emerging themes and areas of agreement in and across groups were identified, as were trends in the number of participants who made the same comment and the intensity or personal context associated with each.

## Results

Most participants were female, used some form of government assistance, and had access to the Internet (Table). Social networking sites (eg, Facebook) were the most commonly used application among participants, mainly as a method of staying in contact with friends and family. Regular use of these sites was reported to be low, which was not due to lack of access but rather lack of time, given participants' busy schedules or other priorities while online. One participant stated, "[I have an account] on Facebook, and I really don't have time to go to it." Some participants had access only in the community (eg, at the public library), where time limits were placed on users.

The results were mixed on whether participants would use social media as a way to obtain information about their children's health. Although some participants were open to exploring a new option, many more objected, citing lack of credibility most often. Participants preferred to obtain health information face-to-face from someone they trusted, particularly when the information concerned the health of their children. According to a participant,

I'm not sure I would use [social media], because I would have to trust [the] person. If they are talking about my kids' health, I don't want some stranger on the computer telling me they need this, and this, and this.

Several participants stressed the desire to consult their doctors, because of their difficulty in trusting online information. However, when participants were asked how they would feel if the social media sites were run by university, government, or nonprofit organizations, they were more amenable to the idea. One participant stated, "It might be a bit more trustworthy than taking someone's information that is just kind of out there."

## Discussion

Although their frequent use of it was low, participants did use social media; therefore, it has value as part of an overall communication strategy that includes more traditional channels. Our results correspond with current trends in social media use, which have shown an increasing trend in the number of people who use social media applications (10-11). Nevertheless, to employ this new channel for information dissemination, barriers (ie, lack of time to use social media and perceived lack of integrity of health information found there) must be overcome.

Our study had several limitations. The sample size was small, so the results are less generalizable to the overall population. Nonetheless, we found several clear themes that can be applied in the context of other research. Another limitation is that participants were selected from a convenience sample from 1 region in Texas; however, our sample was comparable to others that may have been selected from other low-income, minority communities (12-14). Finally, although Hispanic representation in the sample was good, focus groups were conducted in English; therefore, our findings may not apply to Spanish-speaking low-income parents. Further research is needed in this population.

Health messages delivered to low-income parents must come from perceived experts and should be personalized, which may help establish a relationship between information provider and seeker and overcome the barrier of lack of credibility and trust. Parental time is a valuable resource, and social media outlets are most often used by parents as a means for maintaining personal relationships rather than as sources of information. More effort, investment, and creativity are needed to draw this audience to social media sites that contain health information. Future research should focus on ways to most effectively use this new channel of communication in low-income populations, which will require larger-scale quantitative investigation of the trends identified in this exploratory investigation.

## Figures and Tables

**Table.** Demographic Characteristics of Focus Group Participants, Texas, October 2010

**Characteristic^a^**	No. of Participants (N = 19)
**Female sex**	18
**Race/ethnicity**	
White	10
Hispanic/Latino	8
African American	1
**Language(s) spoken at home**	
English	16
English/Spanish	3
**Highest level of education**	
Some college	9
GED/high school diploma	6
Associate degree	1
Bachelor's degree	1
Graduate/professional degree	1
Less than high school diploma	1
**Government assistance^b^**	
No assistance	7
Assistance	12
**Computer at home**	
Yes	13
No	6
**Access to Internet**	
Yes	14
No	5
**Mode of Internet access^c^**	
Computer	13
Cellular telephone	7
Other	5
**Newest Vital Sign test score^d^**	
Unlikely to have low health literacy	9
Likely to have low health literacy	10

Abbreviation: GED, general educational development.

a Mean age of participants was 36.0 y (standard deviation, 9.4 y).

b Government assistance includes any of the following: WIC (Special Supplemental Nutrition Program for Women, Infants and Children), TANF (Temporary Assistance for Needy Families), CHIP (Children's Health Insurance Program), Medicaid/Texas Health Steps, Medicare, SNAP Food Benefits, free/reduced-price meals at school, and any other government assistance program.

c Participants could respond that they accessed the Internet through more than 1 source, so these numbers may not sum to the sample size. "Other" was selected by participants who indicated they did not have access to the Internet.

d Participants were considered unlikely to have low health literacy if they correctly answered 4 questions.

## References

[1] Golley RK, Hendrie GA, Slater A, Corsini N (2011). Interventions that involve parents to improve children's weight-related nutrition intake and activity patterns — what nutrition and activity targets and behaviour change techniques are associated with intervention effectiveness?. Obes Rev.

[2] Hingle MD, O'Connor TM, Dave JM, Baranowski T (2010). Parental involvement in interventions to improve child dietary intake: a systematic review. Prev Med.

[3] Keatinge D (2006). Parents' preferred child health information sources: implications for nursing practice. Aust J Adv Nurs.

[4] Khoo K, Bolt P, Babl FE, Jury S, Goldman RD (2008). Health information seeking by parents in the internet age. J Paediatr Child Health.

[5] Krueger RA, Casey MA, Wholey JS, Hatry HP, Newcomer KE (2010). Focus group interviewing. Handbook of practical program evaluation.

[6] Texas Education Agency. Direct performance summary report definitions.

[7] (2009). Texas Education Agency. Academic excellence indicator system 2008-2009 district performance report: Del Valle independent school district.

[8] (2010). The University of Texas at Austin, School of Nursing. The Children's Wellness Center.

[9] Weiss BD, Mays MZ, Martz W, Castro KM, DeWalt DA, Pignone MP (2005). Quick assessment of literacy in primary care: the Newest Vital Sign. Ann Fam Med.

[10] (2009). Lenhart A [Internet]. Pew Internet Project data memo: adult and social network websites. Pew Internet & American Life Projec.

[11] Chou WS, Hunt YM, Beckjord EB, Moser RP, Hesse BW (2009). Social media use in the United States: implications for health communication. J Med Internet Res.

[12] Kahlor L, Mackert M, Junker D, Tyler D (2011). Ensuring children eat a healthy diet: a theory-driven focus group study to inform communication aimed at parents. J Pediatr Nurs.

[13] Mackert M, Kahlor L, Tyler D, Gustafson J (2009). Designing e-health interventions for low health literate culturally diverse parents: addressing the obesity epidemic. Telemed J E Health.

[14] Makcert M, Kahlor L, Silva K, Padilla Y (2010). Promoting folic acid to Spanish-speaking Hispanic women: evaluating existing campaigns to guide new development. Women Health.

